# CYCLE OF VICTIMIZATION: CHILD MALTREATMENT, INTIMATE PARTNER VIOLENCE, AND ELDER ABUSE IN A U.S. CHINESE POPULATION

**DOI:** 10.1093/geroni/igz038.2793

**Published:** 2019-11-08

**Authors:** Bei Wang, XinQi Dong

**Affiliations:** 1 Institute for Health, Health Care Policy, & Aging Research, Rutgers University, New Brunswick, New Jersey, United States; 2 Rutgers University, New Brunswick, New Jersey, United States

## Abstract

Victims of violence might have higher risks of revictimization, but this has been insufficiently examined among older populations. The present study used cross-sectional data among 3,157 U.S. Chinese older adults in Chicago, Illinois. Multiple logistic regression analyses were used to examine the relationships among subtypes (psychological, physical/sexual, financial exploitation, caregiver neglect) of child maltreatment (CM), intimate partner violence (IPV), and EM. Violence experiences were positively associated. CM psychological was positively associated with IPV psychological (OR 7.60, 95% CI 4.29-13.45) and EM psychological (OR 3.79, 95% CI 2.20-6.51). CM physical/sexual was positively associated with IPV physical/sexual (OR 1.86, 95% CI 1.02-3.38) and IPV physical/sexual was positively associated with EM physical/sexual (OR 8.54, 95% CI 3.53,20.64). EM financial exploitation was positively associated with all types of CM and IPV, whereas EM caregiver neglect has no significant association with any CM or IPV. Clinical and policy implications of the findings will be discussed.

